# Decreasing muscle performance associated with increasing disease activity in patients with rheumatoid arthritis

**DOI:** 10.1371/journal.pone.0194917

**Published:** 2018-04-09

**Authors:** Toini I. Uutela, Hannu J. Kautiainen, Arja H. Häkkinen

**Affiliations:** 1 Department of Medicine, Central Hospital of Lapland, Rovaniemi, Finland; 2 Medical Research Center Oulu, Oulu University Hospital, Oulu, Finland; 3 Unit of Primary Health Care, Helsinki University Hospital, Helsinki, Finland; 4 Unit of Primary Health Care, Kuopio University Hospital, Kuopio, Finland; 5 Department of Physical and Rehabilitation Medicine, Central Hospital of Jyväskylä, Jyväskylä, Finland; 6 Department of Health Science, University of Jyväskylä, Jyväskylä, Finland; Public Library of Science, UNITED KINGDOM

## Abstract

**Objectives:**

Increasing evidence suggests that inflammation has a detrimental effect on muscle strength. Our objective was to analyse the association between muscle performance and different disease activity levels in patients with rheumatoid arthritis (RA).

**Method:**

A total of 199 consecutive outpatients were subject to cross-sectional assessment. Measurements of grip strength, endurance of the upper and lower limbs and trunk strength were combined as a muscle performance composite score (MPCS), using a standardised method. The disease activity for 28 joints (DAS28), radiographs of small joints (Larsen score), rheumatoid factor, body mass index (BMI), comorbidities and anti-rheumatic drugs were verified. Patients’ questionnaires included sociodemographic information, pain level, global disease activity, the Beck Depression Inventory, the mental and physical component scores of Short Form-36 and physical activity level.

**Results:**

Of the 199 patients, 36%, 17% and 47% patients had remission, low/moderate and high DAS28, respectively. The patients in remission had significantly shorter disease duration, better parameters in terms of pain, physician’s assessment, Larsen, Beck or physical component score of Short Form-36, and they were more physically active than other patients. After adjustments for age, sex, RA duration, radiographs and BMI, the decreasing MPCS associated linearly with the increasing DAS28 activity levels (linearity, P <0.001).

**Conclusion:**

Poorer MPCS is clearly associated with higher disease activity in patients with RA. Muscle performance is a modifiable risk factor. The findings suggest evaluating muscle performance in clinical practice as a part of patient care.

## Introduction

RA is predominantly characterised by joint inflammation, but is associated also with changes in body composition [1]. The loss of muscle mass and concomitant increase in fat mass, that is, rheumatoid cachexia, with normal or increased body weight are common features in patients with RA [1]. Loss of muscle mass is an important contributor to loss of muscle strength in RA patients [2], and RA may accelerate age-related sarcopenia which is characterised by low muscle mass, low muscle strength and low physical performance [3]. In patients with RA gradual loss of muscle strength is more pronounced than in the general population of similar age; it is comparable to that in the oldest members of the general population [4]. Decreased muscle strength seems to be visible even during the first months of RA [5]. In patients with longstanding RA, there is lower muscle mass, decreased muscle strength and poorer muscle quality in terms of greater intramuscular fat content than in healthy controls [6]. Physical inactivity in the RA population is common [7], adding to the risk of poor muscle function [2]. Physical inactivity is one of the key mechanisms affecting skeletal muscle mass and body composition, leading to progressive muscle loss and abdominal fat gain [3].

Skeletal muscle tissue is an active endocrine organ accounting for about 40%-50% of the total weight of the human body [3]. Many catabolic factors, such as aging, physical inactivity and inflammation, effect changes in skeletal muscles [3]. The production of tumour necrosis factor-alpha (TNF-alpha) and other cytokines which are critical to the pathogenesis of RA [8] are reported to have catabolic effects on muscle [9]. TNF-alpha and its soluble receptors have been especially associated with a decline in muscle mass and muscle strength in older people in a study with 5-year follow-up [9]. Patients with RA, compared with young and elderly healthy controls, have higher rates of protein breakdown, which correlates with TNF-alpha production along with hormonal mediators [10].

Muscle strength and endurance are determinants of muscle performance. Relatively little is known about how muscle performance relates to RA clinical variables and muscle performance is not routinely assessed in clinical practice in patients with RA. Decreased muscle strength has negative outcomes in RA, and this decreased muscle strength has been reported to associate with disease activity, radiological damage and disability [11,12]. We conducted a cross-sectional study to assess muscle performance in patients with RA with different disease activity levels. In addition to RA-related markers, we examined comorbidities, several behavioural (physical activity, habit of smoking, obesity), sociodemographic and psychological factors as potential contributors. These factors have been linked to muscle strength and /or RA activity in earlier studies [13, 14] but have not been studied before in the context of muscle performance and RA activity.

## Materials and methods

### Study design and subjects

The study is one part of our clinical survey of RA, and it was performed in the Department of Medicine, Central Hospital of Lapland, in Northern Finland. The 230 consecutive patients, aged > 18 years, who met the 1987 revised American College of Rheumatology classification criteria for RA [15], and had an appointment for a routine outpatient rheumatology visit between December 2011 and June 2012, were invited to participate in the study through a mail query. Together with the invitation, the patients received self-report questionnaires, which they were asked to complete 1 to 3 days before attending the clinic, and to take them to the visit. The patients were allocated to 3 groups according to the Disease Activity Score for 28 joint counts (DAS28): DAS28 < 2.6 indicated remission, DAS28 > 2.6 < 5.1 low/moderate activity and DAS28 > 5.1 high disease activity [16].

The study was approved by the Ethics Committee of Oulu University Hospital, Finland. Written informed consent was obtained from all individual participants included in the study.

### Measurement of muscle performance

We used muscle performance field tests convenient in a clinical practice (17). Physiotherapists, who were unaware of the patients’ clinical and patient-reported outcome (PRO) data, performed the tests for muscle performance on the same day that the patients saw the rheumatologist. The tests included grip strength of each hand measured by the Jamar dynamometer, endurance tests of upper and lower limbs, and repetitive arch-up and sit-up tests, according to Alaranta et al [17]. Grip strength was measured in a sitting position, and the elbow was flexed at 90°, with the shoulder in 0° of flexion. The best of 3 trials of each hand was used to calculate the mean values for the right and left hand. The endurance strength of the lower extremities was assessed by squats and the endurance of the upper arms was assessed by the alternate single-arm dumbbell press (women with 5 -kg weights, men with 10 -kg weights). In the repetitive sit-up test for the trunk flexors, each subject was positioned in the supine position, with the knees flexed at 90° and the feet held by a belt on the test bench. The subject raised the upper body and touched the kneecaps with his/her wrist. In the repetitive arch-up test for the trunk extensors, each subject was positioned in a prone position, with the arms along the side, inguinal region at the edge of the test bench, the upper trunk flexed downward at 45° and the feet held in the ankle region. The subject brought the upper trunk up to the horizontal position and back down again. In the above repetitive tests, the movement was repeated as many times as possible with constant speed where one repetition took 2–3 seconds depending on the test.

### Measurement of physical activity

Information on current leisure time physical activity levels was obtained using the Frequency Intensity Time (FIT) Index of Kasari [18]. The FIT Index consists of the following 3 questions: How many days per week do you exercise (5 alternatives from 6 or 7 times per week [5 points] to less than once per month [1 point])? In what type of physical activities do you participate (5 alternatives from high intensity activities resulting in heavy breathing and perspiration [5 points] to very light aerobic activities [1 point])? How many minutes do you work-out (4 alternatives from over 30 minutes [4 points] to under 10 minutes [1 point])? The FIT Index is calculated by multiplying FxIxT. The score range is 1–100, with the points < 36, 37–63 and > 64 indicating low, moderate and high physical activity levels, respectively [18]. In addition, the reported activities were computed by weighting each type of activity by its energy requirements defined in metabolic equivalent task-minutes per week (MET-min/wk) (modified from the International Physical Activity Questionnaire) [19]. Patients participating in activities of under 600 MET-min/wk were classified as having a low activity level, those participating in 600 to 2999 MET-min/wk were classified as having a moderate activity level and those participating in ≥ 3000 MET-min/wk were classified as having a high level of physical activity [19].

### Measurement of clinical and patient- reported outcomes

The health- related quality of life (HR-QoL) was assessed using the Short Form 36 (SF-36) instrument, which includes 8 domains, scored from 0 (worst) to 100 (best) [20]. The SF-36 domains were divided into the physical component score (PCS) and the mental component score (MCS) [20]. Pain and global RA activity were assessed using a 0–100 mm visual analogue scale (VAS), and the depressive symptoms were assessed with the Beck Depression Inventory (BDI) [21].

The patients were examined by one of two rheumatologists (TU, SS). The RA-related factors verified from the medical records included RA duration, presence of rheumatoid factor (RF) and the use of conventional and biological disease-modifying antirheumatic drugs (DMARDs) and prednisolone. The presence of RF was evaluated as positive or negative according to reference values of the hospital at any time over the course of the disease. The duration of RA was defined from the time point at which the criteria for RA were fulfilled according to the rheumatologist and the diagnosis was confirmed. Disease activity was assessed by the DAS28 which included 28 joint counts (metacarpophalangeale and proximale interphalangeale joints, wrists, elbows, shoulders, knees) for both tenderness and swelling (TJC28, SJC28), the erythrocyte sedimentation rate (ESR), and the patient's self- report global activity on the VAS. The index of the DAS28 is calculated based on the following formula: DAS28 = 0.56x√ (TJC 28) +0.28x√ (SJC 28) +0.70xlog_nat_(ESR)+0.014xVAS [22]. Hand and foot X-rays were graded using the Larsen score [23]. The scale was from 0 to 210. Joints that had undergone joint replacements or arthrodesis were scored as grade 5. Comorbidities were assessed using the Charlson Comorbidity Index [24], which assigns 19 somatic diseases with weights of 1, 2, 3 and 6, and is calculated by summing the weights of the conditions. Metabolic syndrome (MetS) was defined by the updated criteria of the National Cholesterol Education Program Adult Treatment Panel III (ATP III) [25]. At the visit, the rheumatologist inquired about current smoking habits.

### Statistical analyses

Muscle performance composite score (MPCS) was obtained by using a standardised method of combining the muscle strength and endurance measurements (grip strength test, dynamic lifting test of upper limbs, repetitive arch-up test, repetitive sit-up test and squat- test). First, each measurement was standardised with a mean of 0 and a standard deviation (SD) of 1, according to gender, and then the mean of the scores was calculated from the available measurements. The data were presented as means with SDs or as counts with percentages. Statistical comparisons were made using the chi-square test (categorical variables), Fisher-Freeman-Halton test (dichotomous variables) and analysis of variance (ANOVA) (continuous variables). Statistical significance for hypotheses of linearity according to DAS28 groups was evaluated using analysis of co-variance (ANCOVA); age, sex, RA duration, Larsen score and body mass index were added to the model as covariates. The MPCS was adjusted for age, sex, RA duration, Larsen score and body mass index. The bootstrap method was used when the theoretical distribution of the test statistics was unknown or in the case of violation of the assumptions (e.g. non-normality). The Stata 14.0, StataCorp LP (College Station, TX, USA) statistical package was used for the analyses.

## Results

### Characteristics of the patients

Twenty-three patients refused to participate, and 8 were excluded (of these, 4 were unable to complete in their questionnaires due to comorbidities, one was not a Finnish speaker, 2 participated in a randomized controlled trial, and one had missing DAS28 data). Of the 199 eligible patients, 36%, 17% and 47% patients had remission, low/moderate and high DAS28, respectively.

The patients in remission had significantly shorter disease duration, better disease parameters in terms of pain, physician’s assessment and Larsen score than the patients with higher activity levels. A higher percentage of biological DMARDs were in use, especially in the patients with low/moderate activity whereas prednisolone was used as a standalone drug, especially in the high activity group. The patients in remission had lower BMIs, reported fewer depressive symptoms and had a better FIT Index and a better physical component score for the SF-36 than did those with higher DAS28 levels. The patients in remission were more physically active compared with other disease activity groups. However, in all groups the activity levels were low according to the FIT Index. The MET values were moderate in the patients with remission and low in the patients with low/moderate and high activity levels (Table 1).

**Table 1:** Demographics, RA-specific characteristics, comorbidities, lifestyle factors and health-related quality of life in patients with RA according to disease activity levels.

Variable	DAS28 activity level			P-value
	Remission N = 72	Low/Moderate N = 33	High N = 94	
Number of females, %	45 (63)	28 (85)	80 (85)	0.009
Age (years), mean (SD)	59 (12)	60 (11)	59 (13)	0.79
Disease duration (years), mean (SD)	7.5 (9.1)	14.1 (12.4)	13.9 (13.0)	<0.001
Rheumatoid factor, n (%)	44 (62)	20 (61)	57 (61)	0.99
Pain (VAS, mm), mean (SD)	24 (19)	43 (23)	59 (24)	<0.001
Physician´s assessment of disease activity (VAS, mm), mean (SD)	7 (9)	18 (12)	32 (20)	<0.001
Larsen score, mean (SD)	17 (36)	48 (60)	49 (62)	<0.001
Current antirheumatic drugs, n (%)	7 9	18 12		0.017
None	1 (1)	1 (3)	4 (4)	
Conventional DMARD	55 (76)	17 (52)	61 (65)	
Biological DMARD	16 (22)	14 (42)	21 (22)	
Only prednisolone	0 (0)	1 (3)	8 (9)	
Body Mass Index, mean (SD)	26.6 (4.3)	26.3 (6.3)	28.4 (5.5)	0.039
Metabolic syndrome, n (%)	30 (42)	15 (47)	48 (52)	0.45
Beck Depression Inventory, median (IQR)	6 (3, 11)	5 (3, 8)	9 (4, 16)	0.003
Charlson Comorbidity Index, mean (SD)	1.5 (0.8)	1.6 (1.2)	1.7 (1.1)	0.47
MET-min /wk, mean (SD)	617 (576)	435 (410)	453 (478)	0.096
Physical activity level (FIT index), mean (SD)	36 (20)	30 (17)	28 (20)	0.037
Current smoking, n (%)	13 (18)	5 (15)	24 (26)	0.32
36-Item Short-Form Health Survey, mean (SD)				
Physical component score	41 (9)	34 (9)	29 (9)	<0.001
Mental component score	53 (10)	55 (9)	51 (12)	0.050

*RA*, rheumatoid arthritis; *DAS28*, Disease Activity Score using 28 joint counts; *SD*, standard deviation; *VAS*, visual analogue scale; *DMARD*, disease-modifying antirheumatic drug; *IQR*, interquartile range; MET-min/wk, metabolic equivalent minutes per week; *FIT*, Frequency Intensity Time.

### Muscle performance measurement and disease activity

**Fig 1:**
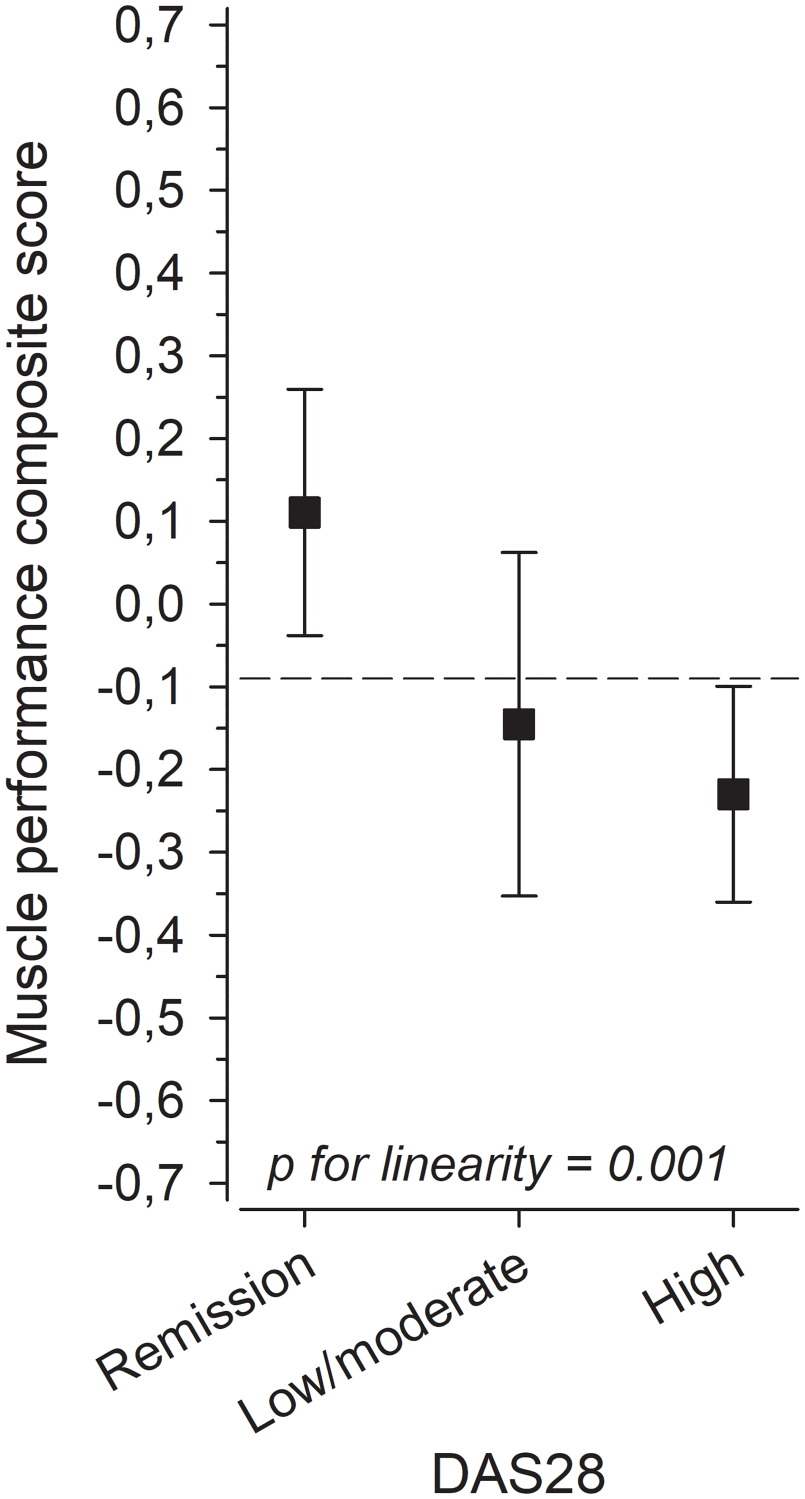
Adjusted (age, sex, RA duration, Larsen score and body mass index) muscle performance composity score according to remission, low/moderate and high DAS28 activity. Values are means with 95% confidence intervals.

The results of the grip-strength test were available for 193 patients, the dynamic lifting test of upper limbs for 151 patients, the repetitive arch-up test for 159 patients, the repetitive sit-up test for 174 patients and the squat -test for 149 patients. The decrease of patients’ MPCS associated linearly with the increase in DAS28 activity levels after adjustments for age, sex, RA duration, Larsen score and BMI (linearity p <0.001) (Fig 1).

With respect to single muscle measurements, after adjustments for age, sex, RA duration, Larsen score and BMI, a linear decrease with increasing DAS28 activity levels was found for grip strength, dynamic lifting test of upper limbs and repetitive sit-up test, but not for repetitive arch-up test and squat-test (Table 2).

**Table 2:** Muscle performance measurements in patients with RA according to different disease activity levels.

Variable	DAS28 activity levels			P-value*
	Remission Mean (SD)	Low/Moderate Mean (SD)	High Mean (SD)	
Grip strength, kg	33.4 (12.8)	23.9 (14.5)	21.4 (11.2)	<0.001
Dynamic lifting test of upper limbs	15.8 (12.8)	15.7 (12.8)	10.3 (11.0)	0.002
Repetitive arch-up test	22.5 (12.9)	18.1 (12.7)	19.2 (15.9)	0.46
Repetitive sit-up test	17.7 (13.4)	14.2 (9.9)	11.8 (10.4)	0.035
Squat-test	17.3 (14.8)	10.9 (11.1)	9.8 (10.4)	0.058

*RA*, rheumatoid arthritis; *DAS28*, Disease Activity Score using 28 joint counts.

* Statistical significance for the hypothesis of linearity according to DAS28 levels were evaluated using the analysis of co-variance (ANCOVA); age, sex, RA duration, Larsen score and body mass index were added to the model as covariates.

## Discussion

The present study shows that the decreasing MPCS is linearly associated with increasing DAS28 activity level after adjustment for important potential confounders such as age, sex, RA duration, Larsen score and BMI.

The physiological decline of skeletal muscle associated with low muscle strength and low physical performance is associated with the aging process [3]. Twenty years ago, Stucki et al observed that in patients with RA, the muscle strength index consisting of the measurements of extension and flexion of knee and elbow joints with a pull gauge correlated moderately with single activity parameters and with DAS [11]. In a recent study by Sheehy et al [26], grip strength alone discriminated between various disease activity states in early RA. Our findings are in line with these reports, indicating decreasing physiological reserves. In practice, decreased muscle strength in RA patients has been associated with worse physical performance and difficulties in carrying out daily activities, both of which may threaten independent living [12].

In our study, all patients had low physical activity levels. The patients with low/moderate and high DAS28 also had low MET values. In patients with RA, energy expenditure is altered compared with healthy people. Many factors such as increased protein catabolism, disease activity, fatigue and physical activity contribute to energy metabolism in patients with RA [10, 27, 28]. In particular, low physical activity seems to be associated with reduced total energy expenditure in patients with RA [27, 28]. Treatment for RA involves active DMARD use to suppress inflammation [16]. Among non-pharmacological treatments, exercise has multiple health benefits for patients with RA and has been suggested as a part of RA patients’ routine care [2]. For instance, regular physical activity 5 years before the RA diagnosis was associated with milder disease at diagnosis in terms of DAS28 and pain [29]. For patients with early RA, a 2-year strength training program was found to improve muscle strength and, additionally, to reduce disease activity. These beneficial effects were maintained throughout the 5-year follow-up period [30]. Disease activity can decrease during intensive exercise, especially in patients with high disease activity [31]. Exercise can modulate cytokine production so that skeletal muscles stimulate the production and release of myokines by contraction, thus reducing the proinflammatory burden which is associated with physical inactivity [32]. In addition to physical inactivity, MetS—a cluster of cardiovascular risk factors [25]–was common in our patients. Cardiovascular diseases, which are recognised to a large extent as low-grade inflammatory disorders, are well-known comorbidities in RA [32, 33]. Physically inactive patients with RA have significantly higher cardiovascular risk profiles than physically active patients [33]. Exercise seems to reduce cytokine levels in patients with ischaemic heart diseases [32]. Thus, physical inactivity in our patients may indicate added risk for cardiovascular comorbidity.

The present study shows that muscle performance is an important outcome variable in RA and has to be taken into consideration in patients’ care. The RA management guidelines recommend the use of exercise and physical activity for patients to reduce symptoms and long-term consequences of RA, but they do not include details regarding physical activity [34]. Lack of clarity and specificity about physical activity guidelines may impact patients’ physical activity behaviour so that patients remain less physically active [34]. Matschke et al [35] have suggested that rheumatoid muscle has the potential to respond to physical training in a similar way to healthy muscle. They found in their physiology study that muscle specific force and architecture were preserved in stable RA patients despite impaired physical function, compared with healthy controls. Sharif et al [36] have reported in their case study that in patients with RA, 16- week strength training increases the size of type I and type II muscle fibres thus improving muscle structure and muscle function. Combined strength and endurance training have increased the strength of the lower extremities and aerobic performance capacity in women with RA and stable disease during a 21 week-training period [37]. Thus, broader muscle performance testing than only grip strength is advisable in the rehabilitative management of RA when counselling and monitoring patients. In the light of our study, prospective studies are needed to investigate how exercises involving the combination of endurance and strength training may improve disease activity and impact the disease course of RA over the long-term. In routine practice, rheumatology staff should motivate patients to implement a more physically active lifestyle.

The main limitation of the present study is its cross-sectional design, which does not justify conclusions on causality. We have no data on why certain patients did not complete different strength tests. Due to missing performance tests, we developed the MPCS. There are many methods to assess muscle performance. The repetitive and endurance tests used in our study have been reported to be fairly reliable [17]. The accuracy of self-reported physical activity compared to that of direct measurements can be debated; however, to date, there is no formal consensus on a ´´´correct´´´- method for defining or describing levels of physical activity on the basis of self- reports [19]. Our patients’ self-reports of physical activity levels were quite aligned with their MET values. The strength of our study is that our patients represent the whole spectrum of RA, from mild to very severe, with patients of variable disease durations and different ages. We also had access a great variety of demographic, RA-related and other clinical covariates and lifestyle factors which can potentially impact muscle strength and/or disease activity according to the literature [1, 3, 4, 11–14]. The study was carried out in a single outpatient clinic, but we consider that our patients represent typical Finnish clinic-based RA patients. According to the prevailing national Care Guidelines [38], our study comprises patients with an early phase and those with a more active disease in an established phase. In addition, selection bias of patient recruitment into the study is minimal because our clinic is the only centre which provides rheumatological services for the district.

## Conclusion

The findings of the present cross-sectional study suggest that the MPCS has a linear association with DAS28 activity. Muscle performance and physical activity are modifiable risk factors. Prospective studies are needed to establish the association between muscle strength and disease activity in patients with RA.
